# HIV-1/HSV-2 Co-Infected Adults in Early HIV-1 Infection Have Elevated CD4+ T Cell Counts

**DOI:** 10.1371/journal.pone.0001080

**Published:** 2007-10-24

**Authors:** Jason D. Barbour, Mariana M. Sauer, Elizabeth R. Sharp, Keith E. Garrison, Brian R. Long, Helena Tomiyama, Katia C. Bassichetto, Solange M. Oliveira, Maria C. Abbate, Douglas F. Nixon, Esper G. Kallas

**Affiliations:** 1 HIV/AIDS Division, San Francisco General Hospital, University of California San Francisco, San Francisco, California, United States of America; 2 Department of Experimental Medicine, University of California San Francisco, San Francisco, California, United States of America; 3 São Paulo City Health System, São Paulo, Brazil; 4 Division of Infectious Diseases, Federal University of Sao Paulo, São Paulo, Brazil; New York University School of Medicine, United States of America

## Abstract

**Introduction:**

HIV-1 is often acquired in the presence of pre-existing co-infections, such as Herpes Simplex Virus 2 (HSV-2). We examined the impact of HSV-2 status at the time of HIV-1 acquisition for its impact on subsequent clinical course, and total CD4+ T cell phenotypes.

**Methods:**

We assessed the relationship of HSV-1/HSV-2 co-infection status on CD4+ T cell counts and HIV-1 RNA levels over time prior in a cohort of 186 treatment naïve adults identified during early HIV-1 infection. We assessed the activation and differentiation state of total CD4+ T cells at study entry by HSV-2 status.

**Results:**

Of 186 recently HIV-1 infected persons, 101 (54 %) were sero-positive for HSV-2. There was no difference in initial CD8+ T cell count, or differences between the groups for age, gender, or race based on HSV-2 status. Persons with HIV-1/HSV-2 co-infection sustained higher CD4+ T cell counts over time (+69 cells/ul greater (SD = 33.7, p = 0.04) than those with HIV-1 infection alone (Figure 1), after adjustment for HIV-1 RNA levels (−57 cells per 1 log_10_ higher HIV-1 RNA, p<0.0001). We did not observe a relationship between HSV-2 infection status with plasma HIV-1 RNA levels over time. HSV-2 acquistion after HIV-1 acquisition had no impact on CD4+ count or viral load. We did not detect differences in CD4+ T cell activation or differentiation state by HSV-2+ status.

**Discussion:**

We observed no effect of HSV-2 status on viral load. However, we did observe that treatment naïve, recently HIV-1 infected adults co-infected with HSV-2+ at the time of HIV-1 acquisition had higher CD4+ T cell counts over time. If verified in other cohorts, this result poses a striking paradox, and its public health implications are not immediately clear.

## Introduction

HIV-1 is often acquired in the presence of pre-existing co-infections, such as Herpes Simplex Virus 2 (HSV-2). The effects of co-infection on clinical course of HIV-1 infection may be complex and vary by the co-infecting agent. Many reports suggest an increase in the risk of HIV-1 transmission to HSV-2 infected individuals, reviewed in [1], with recent reports suggesting possible molecular mechanisms [2]. HSV infection has been shown to increase HIV-1 replication in some studies [3], but has shown no association in other studies [4].

The impact of HSV-2 infection on the human immune system and its ability to counter other infections such as HIV-1 may be complex [5]. HSV-2 infection alters the function and phenotype of monocytes [6]–[8], impeding their maturation, which may in turn alter the phenotype and function of interacting CD4+ and CD8+ T cells. HSV-2 is a ligand for certain TLR molecules [9], and may mediate the effect on dendritic cells. HSV-2 may alter the innate immune system, with subsequent impacts on the adaptive immune system.

We examined the impact of HSV-2 status at the time of HIV-1 acquisition on subsequent clinical course. In agreement with others, and in contrast to several other groups [3], [10], we observed no effect of HSV-2 status on viral load [4]. We observed that treatment naïve, recently HIV-1 infected adults co-infected with HSV-2+ at the time of HIV-1 acquisition had higher CD4+ T cell counts over time. This effect was independent of viral load and other common co-infections. These results suggest a pre-existing HSV-2 infection may directly modulate the CD4+ T cell population during early HIV-1 disease, with possible beneficial effects on clinical outcomes.

## Methods

### Cohort and Laboratory Measures

We examined CD4+ and CD8+ T cell counts, plasma HIV-1 RNA levels in a cohort of 186 treatment naïve adults identified during early HIV-1 infection (study entry within 170 days of HIV-1 seroconversion) by STARHS [11]. These individuals were enrolled and followed at the Federal University of Sao Paulo, Brazil, and removed from the analysis at the time they started anti-retroviral therapy or were lost to follow-up. HIV-1 subtypes were predominantly B and F, with some circulating recombinant forms [12]. We collected information on participant age, gender, race and CCR5 delta32 genotype. In addition, we determined Hepatitis B, GBV-C and HSV-2 co-infection serology by indirect ELISA assays (GBV-C ELISA, provided by Dietmar Zdunek, Roche Diagnostics, Germany; HSV-2 ELISA. Dia Sorin, Saluggia, Italy). We employed Diasorin ‘Method 2’ which is an ELISA based method with high sensitivity to detect primary, secondary and recurrent HSV-2 infections but has lowered specificity [13]. We assessed the activation state (CCR5 percent and CD38 percent or MFI, both by Parmingen, San Diego, CA) and the differentiation state (CCR7, CD25, CD28 and CD27, all from BD Biosystems, San Jose, CA) of total CD4+ T cells by four color flow cytometric panels in a FACSCalibur (BD Biosystems), followed by analysis using Cell Quest software (BD). We did not detect differences in T cell activation state, nor differentiation state of total CD4+ T cells by HSV-2+ status. Ethical approval was obtained from the Federal University of Sao Paulo IRB and patients gave informed consent.

### Statistical Analysis

As is standard clinical practice in Brazil, subjects are not started on antiretroviral drug therapy until CD4+ T cell counts have decreased to 350 CD4+ T cells/µl or below, or is otherwise clinically indicated. In this study, we right censored (removed from analysis) subjects at the time they initiated antiretroviral therapy, which was initiated when CD4+ T cells fell bellow 300 cells/µl. We used longitudinal mixed effects models to assess the relationship of co-infection status on CD4+ T cell counts and HIV-1 RNA levels, with random effects for time and intercept. We used the Chi Square or Fisher's Exact to test differences in proportions between the two groups by HSV-2 status, and the Wilcoxon Two Sample Test to compare differences in continuous variables at study entry. All analyses were performed in SAS 9.2 for Windows XP.

## Results

Of 186 recently HIV-1 infected persons, 101 (54 %) were sero-positive for HSV-2, and the balance tested negative. A full description of the cohort at study entry status may be found in Table 1. There was no difference between the groups for age, gender, race, or CCR5 delta32 genotype based on HSV-2 status.

**Table 1:** Clinical and Demographic Characteristics of the Study Cohort At Study Entry.

Measure	Median	IQR	N
Log_10_ HIV-1 RNA c/mL	4.3	(3.6, 4.9)	177
CD4+ T cell counts/uL	535	(409, 698)	184
Age (Years)	30.8	(24.7, 36.3)	186
**Clinical Follow-Up**			
Observation Time (Days)	389	(117, 833)	186
Total Person Days (Sum)	96466		186
Time Until ART Started (Days)*	381	(129.5, 649)	49

Measure	Frequency	Number
HSV-2+ Serostatus	54%	101/186
HCV Serostatus	2%	2/115
HBV Serostatus*	39%	45/117
**Demographics**		
Male Gender	92%	168/186
Ethnicity		
Asian	1%	2
White	60%	113
Mulato	19%	34
Black	8%	14
Other	12%	22
Brazilian Amerindian	0.5 %	1

* Anti Hepatitis B Core Antibody.

**Figure 1:**
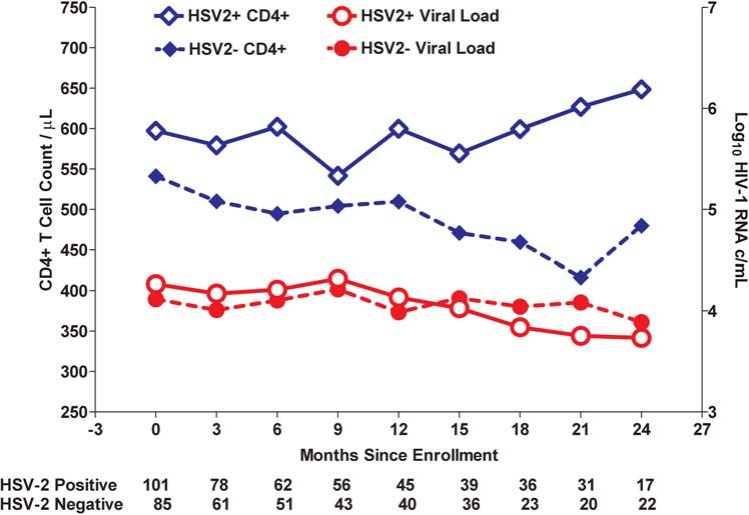
Higher absolute CD4+ T cell counts in HSV-2+/HIV-1+ subjects independent of HIV-1 viral load. The open blue diamonds represent the CD4+ T cell counts, and the open red dots represent HIV-1 viral load over time for persons co-infected with HSV-2 at the time of diagnosis with an early HIV-1 infection. The solid blue diamonds represent the CD4+ T cell counts, and the solid red dots HIV-1 viral load, for those HSV-2 negative at the time of early HIV-1 infection. Observations were right censored at the time anti-retroviral therapy was initiated, which occurred at approximately a CD4+ T cell count of 350 cells/µL.

In a longitudinal mixed effects regression model, we observed that persons with HIV-1/HSV-2 co-infection sustained higher CD4+ T cell counts over time (average of 69 cells/µl greater (SD = 33.7, p = 0.04)) than those with HIV-1 infection alone (Fig. 1), after adjustment for HIV-1 RNA levels (−57 cells per 1 log_10_ higher HIV-1 RNA, p<0.0001). We did not observe a relationship of HSV-2 infection status with plasma HIV-1 RNA levels over time (Fig. 1).

HSV-2 co-infection was associated with positive Hepatitis B core antigen serostatus (Chi Square, p = 0.02). In a statistical model adjusted for hepatitis B core antigen serostatus as well as viral load, HSV-2+ status tended to associate with higher CD4+ T cell counts over time (89 cells higher for HSV2+, p = 0.07), while hepatitis B serostatus did not (69 cells higher for anti-HBc+, p = 0.16). This result suggests the association of HSV-2 status with CD4+ T cell counts is independent of viral load and hepatitis B core antigen serostatus, but does not rule out the possibility that HBV infection (either active infection or cleared infection) may also be associated with higher CD4+ T cell counts in early HIV disease. Likewise, the association of HBV with CD4+ T cell counts may be driven in part or entirely via its association with HSV-2+.

Unlike HSV-2, which is a persistent infection in humans, hepatitis B may be cleared. Hepatitis B core antigen status reflects exposure and may not indicate active infection. Hence, anti-hepatitis B core antigen status likely reflects higher net exposure to sexually transmitted agents, as is reflected in the association of anti-HBc+ correlation with HSV-2 infection. We also examined the rate of positive GBV-C serostatus, and found it was slightly higher in the HSV-2+ than HSV-2– HIV-1 infected groups (27% versus 25%). This difference was not significant and we found no association with CD4+ T cell count or viral load by GBV-C status.

We did not detect an association between study entry HSV-2 status and time to initiation of anti-retroviral therapy. However, as only 48 of the 186 enrolled initiated ART during our period of observation we may have been underpowered to observe such an effect should it exist.

### Incident HSV-2 Infection, CD4+ T cell counts, and HIV-1 RNA Level

**Figure 2:**
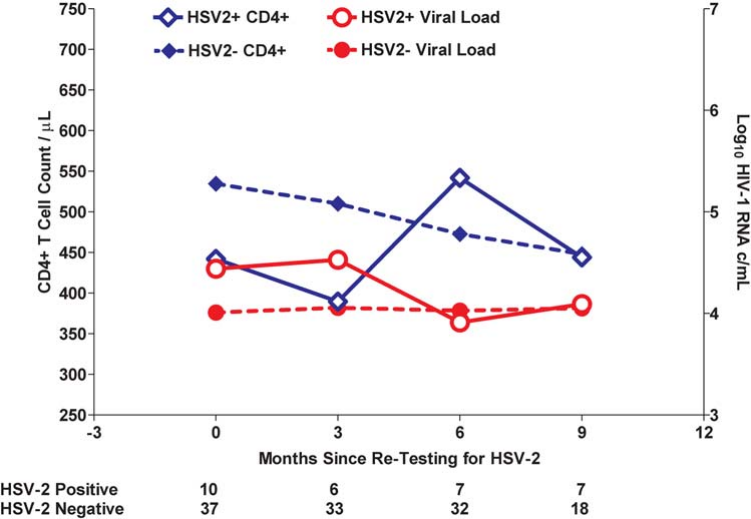
HSV-2 acquisition after HIV-1 acquisition is not associated with subsequent differences in CD4+ T cell counts or HIV-1 viral load. The open blue diamonds represent the CD4+ T cell counts, and the open red dots represent HIV-1 viral load, over time for persons testing positive for HSV-2 at 1 year after study enrollment. The solid blue diamonds represent the CD4+ T cell counts, and the solid red dots HIV-1 viral load, for those HSV-2 negative at 1 year after study enrollment. Forty-seven of the 85 persons who tested HSV-2 negative at study entry were re-tested. Observations were right censored at the time persons initiated anti-retroviral therapy, which was initiated at approximately a CD4+ T cell count of 350 cells/µL.

We measured HSV-2 status after one year of follow-up in a set of 53 persons who had tested negative at study entry. Of 53 persons tested, 47 had sufficient clinical follow-up to allow evaluation of HSV-2 test results on subsequent CD4+ and viral load levels. Of these 47, 10 (21.2 %) had seroconverted to HSV-2+ status since acquiring HIV-1 and entering the cohort. We found that the acquisition of HSV-2 after infection HIV-1 was not associated with subsequent changes in viral load or with CD4+ T cell count during the untreated time period (Fig. 2).

### CD4+ T cell Phenotype and HSV-2+ Status

**Figure 3:**
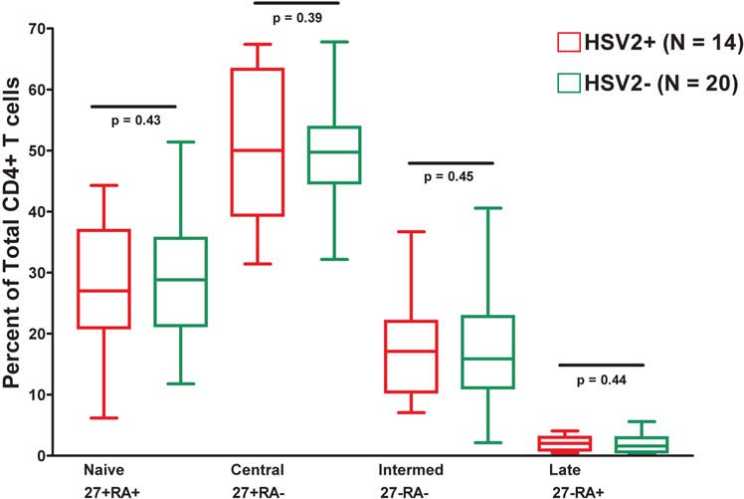
No Difference in Total CD4+ T Cell Phenotypes by HSV-2 Status. Red symbolizes those persons who test positive for HSV-2 at study entry and are HIV-1 co-infected. Green are those persons who tested negative for HSV-2 at study entry and are infected with HIV-1 only. ‘27’ represents CD27, and ‘RA’ represents CD45RA, which were used to define differentiation stages of the total CD4+ T cell pool.

Among 34 recently HIV-1 infected persons, we compared 14 persons HSV-2+ at study entry with 20 HSV-2- persons for differences in total CD4+ T cell phenotype, including activation markers, and differentiation markers (Fig. 3). We observed no difference in the activation state of CD4+ T cells by HSV2+ status, or with the differentiation status of CD4+ T cells.

## Discussion

We found that HIV-1/HSV-2 co-infected treatment naïve adults in early HIV-1 infection had higher CD4+ T cell counts than those infected with HIV-1 alone. This effect may be due to the effects of a pre-existing HSV-2 infection on the immune system, a dynamic which may remain in place after acquisition of HIV-1. The association of HSV-2 with higher susceptibility to HIV-1 infection may be attributable to higher CD4+ T cell counts in circulation and in turn with higher target cell availability for HIV-1 in sites of entry. This effect of HSV-2 on CD4+ T cell counts may further heighten risk of infection beyond the increased opportunity for HIV-1 viral entry through genital lesions caused by HSV-2.

Incident HSV-2 infection – that is seroconversion or acquisition of HSV-2 after infection HIV-1 – was observed in our cohort, and analyzed separately for its effect on viral load and CD4+ T cell count. We did not detect subsequent changes in the CD4+ or viral load among a subset of this cohort who were observed for acquisition HSV-2 subsequent to HIV-1 infection. That an incident HSV-2 infection did not modulate the relationship of HIV-1 to viral load or CD4+ T cell count suggests that the effect of HSV-2 infection on CD4+ T cell counts manifests prior to acquisition of HIV-1.

The manner by which HSV-2 co-infection may confer a protective effect on CD4+ T cell populations is not clear. To explore a link between HSV-2 infection and changes in T cell differentiation we gauged the differentiation stage of total CD4+ T cells. We detected no expansion of any CD4+ T cell differentiation subset between HSV-2+ and HSV-2- persons. HSV-2 may stimulate cytokines which promote new CD4+ T cell production, decrease the rate of CD4+ activation induced T cell destruction or potentially increase CD4+ T cell survival [14]–[17]. Recent evidence suggests that herpesvirus may have broad immunomodulatory effects. In a murine model of gammaherpesvirus (γHV68) infection, latent infection was associated with elevated levels of serum IFN-γ and TNF-α, and protected mice from infection by two distinct bacterial strains [18]. Herpesvirus infection may have a protective effect against other infections by establishing a level of innate immunity that is maintained as the host tries to prevent HSV-2 emergence from latency.

Herpes virus infections, which are highly prevalent among humans, may not be solely pathogenic, and may in fact impart some immunologic benefit to the host [18]. More recent results from our group (Long et al, Submitted 2007) indicate that HSV-2 infection has pan-leukocytic effects, with general increases in both CD4+ and CD8+ T cell counts as well as Natural Killer (NK) cell counts. Additional results from this same study suggest HSV-2 infection may distract or decoy NK cells from targeting HIV-1. Clinical pathology from HSV-2 infection is only observed in a fraction of those with HSV-2. However the burden of disease from HSV-2 and other herpesviruses (EBV, HHV-8, Zoster) is significant worldwide. The theory of HSV-2 viral adaptation conferring benefit to the host must be approached with caution.

Our study is subject to several limitations. Our method for detection of HSV-2 infection is of high sensitivity, indicating a very low false negative rate. However, our method has lower specificity, indicating some individuals counted as HSV-2 infected here may in fact not be carriers. There is continuing debate over the optimal method for detection of HSV-2 status – ELISA based methods such as ours are rapid and highly sensitive but do not match the precision and accuracy of the western blot [13]. The higher false positive rate translates to a conservative bias. That is – the effects of HSV-2 on higher CD4+ counts seen here - are likely diluted, not amplified, by the inclusion of HSV-2 negative persons. Hence, the difference in CD4+ T cell counts by HSV-2 status may in fact be larger than reported here. In addition there may exist a modest difference in viral load in early infection which we did not detect.

There is a growing body of research on the role of HSV-2 in the risk acquisition of HIV-1 [19], [20]. A recent study by Nagot et al. has demonstrated that daily valacyclovir to suppress HSV-2 replication was associated with reductions in genital and plasma HIV-1 RNA levels [3]. Our study differs from that of Nagot et al. in several ways. In our cohort we 1) studied persons identified in very early HIV-1 infection, 2) our study was mostly men (90 %) compared to the Nagot trial which was all female, and 3) valacyclovir was not used to control re-activations of HSV-2 disease (its use in Brazil is uncommon due to expense). Thus our findings represent the impact of the natural history of HSV-2 serostatus on HIV-1 disease markers. Gender may play an important role in modulating the relationship of HSV-2 to immune status during HIV-1 infection. Indeed, results from HSV-2 subunit vaccine trials have indicated that subunit vaccines are immunogenic and confer protection among women, but not men [21]. The basis for the role of gender in modulating this response is not clear but may at least partially explain differences between studies discussed here.

Worldwide, HSV-2 is a common infection in adults [22], and among persons with HIV-1 or at risk for HIV-1 infection the prevalence of HSV-2 may be greater than 50 %. Having HSV-2 at the time of HIV-1 acquisition appears to have no effect on plasma viral load during early infection, yet may be associated with higher CD4+ T cell counts. HSV-2 acquisition after acquisition of HIV-1 was not associated with subsequent differences in CD4+ T cell counts or HIV-1 RNA level. This suggests the order in which HSV-2 and HIV-1 are acquired may influence the nature of HSV-2 impact on the immune system.

Hence, the same viral co-infection which may increase susceptibility to HIV-1 infection due to chronic mucosal tissue inflammation and or ulceration may in turn be associated with higher CD4+ T cell counts after acquisition of HIV-1. This is a striking paradox, and its public health implications are not immediately clear. It will be of interest to determine if other cohorts of recently infected adults display a similar relationship of HSV-2 co-infection with elevated CD4+ T cell counts over time. Such an association may have been overlooked in favor of examination of an association with viral load. Recent reports have suggested that HIV-1 viral load accounts for a relatively small share of the rate of CD4+ T cell loss in HIV disease [23]. HSV-2 co-infection may account for some of the variation in that relationship by boosting CD4+ T cell counts in early infection, in a manner independent of viral load.
